# Clinical impact of the Development of Communication Skills in Autism (DHACA) on the development of social skills in children with autism

**DOI:** 10.1590/2317-1782/e20250202en

**Published:** 2026-08-03

**Authors:** Letícia Cristiny Arcanjo da Silva, Fádja Auxiliadora Alves e Silva, Rafaela Asfora Siqueira Campos Lima, Ana Cristina de Albuquerque Montenegro

**Affiliations:** 1 Departamento de Fonoaudiologia, Universidade Federal de Pernambuco – UFPE - Recife (PE), Brasil.; 2 Programa de Pós-Graduação em Saúde da Comunicação Humana, Departamento de Fonoaudiologia, Universidade Federal de Pernambuco –UFPE - Recife (PE), Brasil; 3 Departamento de Psicologia, Inclusão e Educação – DPSIE, Universidade Federal de Pernambuco – UFPE - Recife (PE), Brasil.

**Keywords:** Autism Spectrum Disorder, Social Skills, Augmentative and Alternative Communication, Language, Speech Therapy

## Abstract

**Purpose:**

To evaluate the clinical impact of the DHACA® method on the development of social skills in children with Autism Spectrum Disorder (ASD).

**Methods:**

This longitudinal quantitative intervention study conducted with 23 children with ASD, aged 3 to 6 years, who were nonverbal or minimally verbal. Individual intervention sessions using the DHACA® method were held weekly for 20 weeks. Social skills were assessed before and after the intervention using the ACOTEA-R protocol. Data were statistically analyzed using the Wilcoxon test.

**Results:**

There was a significant improvement (p < 0.05) in ten of the sixteen skills assessed, including “responds to name,” “eye contact,” “shared attention,” and “symbolic play.” Other skills, such as “responds to ‘no’” and “takes turns,” showed a tendency toward clinical improvement, although not statistically significant. Only three skills did not show relevant progress.

**Conclusion:**

The DHACA® method proved effective in promoting social skills in children with ASD, contributing to greater autonomy and social participation. The results reinforce its clinical potential as a therapeutic resource in both educational and healthcare settings.

## INTRODUCTION

Autism spectrum disorder (ASD) is a neurodevelopmental disorder characterized by persistent deficits in communication, difficulties in social interaction, and restricted and repetitive patterns of behavior^(1)^. These characteristics can vary in severity and functioning, manifesting from the first years of life^(2)^. Recent data from the Centers for Disease Control and Prevention (CDC) indicate that ASD affects approximately 1 in 36 eight-year-old children in 11 US states^(3)^, showing a significant increase from previous surveys^(4)^. This growing prevalence reinforces the need for effective interventions aimed at minimizing communication barriers and promoting the social inclusion of individuals with ASD.

Difficulties in social functioning are one of the main defining characteristics of autism^(5)^. Social perception, one of the fundamental components of social skills, refers to the ability to interpret and assign meaning to social stimuli, such as facial expressions, tone of voice, and behaviors. Individuals with ASD have deficits in social perception, which compromise the development of essential skills for social interaction. Furthermore, there are impairments in cognitive skills and social reciprocity, significantly impacting the quality of life of these individuals^(6)^.

Del Prette and Del Prette^(7)^ define social skills as a set of learned behaviors that enable individuals to interact effectively in social contexts, promoting acceptance and inclusion. Studies indicate that some of the main social skills include smiling and maintaining eye contact, initiating and maintaining interactions, sharing attention, inferring interests, and adopting the perspective of others^(5)^. Promoting these skills can be facilitated through innovative approaches, such as augmentative and alternative communication (AAC), which aims to expand the communicative possibilities of individuals with expressive difficulties^(8)^.

AAC has been widely studied as an effective resource to promote communication and social inclusion of individuals with ASD^(9)^. According to the American Speech-Language-Hearing Association (ASHA), AAC comprises techniques, resources, and strategies that facilitate communication for people with complex communication needs (CCN)^(2)^. Among the benefits described in the literature, the following stand out: improvement in facial and body expression, development of oral language, enhancement of nonverbal language, and expansion of social participation^(9)^. These factors help to reduce frustration, minimize inappropriate behaviors, and strengthen interpersonal interactions^(9)^.

The Development of Communication Skills in Autism (DHACA®) is an innovative AAC method, based on Social-Pragmatic Theory or Use-Based Language Acquisition Theory, which emphasizes the role of social interaction in the development of communication. This method uses a communication book with pictograms of core and fringe vocabulary, organized into lexical categories, to promote the acquisition and functional use of language. DHACA® consists of five skills that stimulate the development of functional communication through strategies such as the use of modeling and physical, visual, and verbal cues. In addition, its approach is based on the child's individual interest, making learning more meaningful and motivating. The implementation of DHACA® has shown potential to expand the communicative and social skills of children with ASD, providing greater autonomy, active participation in social interaction, and quality of life^(10)^.

Given the increasing prevalence of ASD and the need for effective strategies for the development of social skills, it is essential to investigate the impact of the DHACA® method on promoting these skills. The scarcity of studies evaluating the relationship between the application of DHACA® and the improvement of social skills in children with ASD reinforces the need for research analyzing its benefits. Thus, this study aimed to evaluate the effects of the DHACA method on the development of social skills in children with ASD.

## METHODS

This longitudinal, quantitative, intervention study was carried out at the Professor Fábio Lessa Speech-Language-Hearing Teaching Clinic, linked to the Speech-Language-Hearing Department of the Federal University of Pernambuco (UFPE). The project was approved by the Human Research Ethics Committee under protocols no. 2.106.800 and no. 6.617.543, respecting the ethical precepts established in Resolution no. 466/2012 of the Brazilian National Health Council.

The research included 23 children with a clinical diagnosis of ASD, participants in the “Autismo Comunica” outreach and research project in 2022 and 2023; four of them were female, and 19 were male. Eight of them were non-speaking, and 15 were minimally speaking, referred to the teaching clinic by the Unified Health System (SUS). The inclusion criteria were age between 3 and 6 years, with a diagnosis of ASD according to the criteria established by the American Psychiatric Association in the DSM-5 (2015), and not undergoing concomitant external speech-language-hearing intervention. Children with a predominantly speaking communicative profile were excluded, as well as those with comorbidities associated with ASD, such as genetic syndromes, malformations, neurological disorders, and physical, intellectual, auditory, or visual impairments.

The children's legal guardians were invited to participate and, after being informed about the study objectives and procedures, signed an informed consent form. Then, a clinical history interview and a sociodemographic questionnaire were conducted to characterize the participants and record their clinical and family history. Regarding the parental education profile, 20 guardians had completed high school, and three had completed elementary school.

The children were assessed before and after speech-language-hearing intervention using the Communication Assessment in ASD protocol (ACOTEA-R)^(11)^, with playful and interactive activities in a therapeutic environment. ACOTEA-R allows the observation of 36 social and communicative skills, distributed among the sections of Expressive Communication, Receptive Communication, and Social Behavior. Among the skills in the instrument, 16 were selected for analysis, prioritizing items whose nature is predominantly social. Skills related mainly to communication and behavioral issues were not included, as they do not constitute direct indicators of sociability. The methodological decision was based on developmental models that describe a hierarchy of initial social competencies, such as shared attention, imitation, and engaged play, considered prerequisites for the emergence of more complex social repertoires^(11-13)^.

Thus, the skills chosen were “offers or shares something” (P11), “performs social productions” (P14), “asks questions” (P15), “respects turns” (P19), “does not throw tantrums” (P20), “social smile” (P21), “responds to name” (P22), “responds to ‘no’” (P24), “understands and executes simple commands” (P25), “imitation” (P26), “shared attention” (P27), “expresses affection” (P28), “eye contact” (P29), “takes initiative to perform activities” (P32), “symbolic play” (P35), and “engaged play” (P36). The ACOTEA-R assessment was recorded as previously authorized by the guardians through a photo consent form. An evaluator blinded to the condition responded to the instrument before and after the intervention.

The intervention with the DHACA® method was conducted in 20 individual, weekly sessions, lasting 45 minutes each. The activities were meticulously planned with playful activities, based on structured and semi-structured games, respecting each child’s preferences, previously identified through the Indirect Preference Assessment (IPA)^(10)^. The instrument, answered by the caregiver, aims to identify the child's preferences in different domains, including food choices, social interactions, sensory stimuli, toys, activities, and objects of interest. Therapeutic strategies involved the use of cues, whether physical (partial or total physical cue), visual (directive gestures and visual resources), or verbal. These elements were incorporated to make the proposals more motivating, facilitate the assimilation of tasks, and encourage more accurate responses from the children^(14,15)^. The gradual reduction of cues was applied individually, according to the performance of each participant, respecting their learning pace.

The interventions were conducted by speech-language-hearing undergraduate and postgraduate students, who had been previously trained at the beginning of the academic year and continuously supervised throughout the process by faculty members from the research and outreach group "Autism Communicates". Supervision occurred in real time using a one-way mirror, in addition to regular meetings for case discussions and review of therapeutic strategies.

The children's caregivers participated in weekly individual guidance sessions during or after the intervention session. They also participated in parent guidance meetings every 2 months, lasting approximately 3 hours each, in which the theoretical bases of the social and communicative skills worked on were discussed, as well as practical strategies for using the DHACA® book in daily life, and moments of listening, sharing, and clarifying doubts. This participation aimed to broaden the family's involvement in the therapeutic process, enabling continuity and reinforcement of interventions in the family environment.

The data collected were organized in digital spreadsheets in Microsoft Excel and subsequently subjected to statistical analysis. Social and communicative skills were analyzed using descriptive measures (mean, standard deviation, median, and interquartile range). As the data did not present a normal distribution, verified by the Shapiro-Wilk test, the Wilcoxon test with continuity correction was applied to compare the scores between before and after the intervention. This test is indicated for paired samples with an asymmetrical distribution, as in the case of the present study.

## RESULTS

The research results indicate that AAC intervention through the DHACA method favored the development of social skills in the participants. There was a statistically significant improvement, evidenced by p-values ​​below 0.05, in several items related to social and communicative skills, as seen in Table 1 below.

**Table 1:** Scores of social skills from the ACOTEA protocol before and after intervention in children with autism, with statistically significant improvement (p < 0.05)

**Skill**	**1. Before, N = 23**	**2. After, N = 23**	**p-value1**
**Responds to the name**			**0.003**
Mean (SD)	1.52 (0.95)	2.52 (0.95)	
Median [IQI]	2.00 [1.00. 2.00]	2.00 [2.00. 3.00]	
**Eye contact**			**0.001**
Mean (SD)	1.91 (0.95)	2.74 (0.96)	
Median [IQI]	2.00 [1.50. 2.00]	3.00 [2.00. 3.00]	
**Shared attention**			**0.003**
Mean (SD)	1.52 (0.79)	2.43 (1.20)	
Median [IQI]	2.00 [1.00. 2.00]	3.00 [1.50. 3.00]	
**Understands and executes simple commands.**			**0.003**
Mean (SD)	1.74 (1.18)	2.78 (1.09)	
Median [IQI]	2.00 [1.00. 3.00]	3.00 [2.00. 4.00]	
**Imitation**			**0.008**
Mean (SD)	1.39 (1.03)	2.13 (1.22)	
Median [IQI]	1.00 [1.00. 2.00]	2.00 [1.00. 3.00]	
**Expresses affection**			**0.014**
Mean (SD)	0.87 (1.06)	1.48 (1.38)	
Median [IQI]	1.00 [0.00. 1.00]	1.00 [0.00. 3.00]	
**Symbolic play**			**0.005**
Mean (SD)	1.35 (1.23)	2.26 (1.48)	
Median [IQI]	1.00 [0.00. 2.00]	3.00 [1.00. 3.00]	
**Offers or shares something**			**0.046**
Mean (SD)	0.57 (0.79)	1.22 (1.41)	
Median [IQI]	0.00 [0.00. 1.00]	0.00 [0.00. 2.50]	
**Social smile**			**0.047**
Mean (SD)	2.00 (1.24)	2.48 (1.20)	
Median [IQI]	2.00 [1.00. 2.50]	2.00 [2.00. 3.50]	
**Executes social productions.**			**0.005**
Mean (SD)	0.48 (0.99)	1.52 (1.41)	
Median [IQI]	0.00 [0.00. 0.00]	2.00 [0.00. 2.00]	

**Caption**: SD – Standard Deviation; IQI – Amplitude Interquartile

Among the skills with significant differences, the following stand out: "**responds to name**" (p = 0.003), "**eye contact**" (p = 0.001), "**shared attention**" (p = 0.003), "**understands and executes simple commands**" (p = 0.003), "**imitation**" (p = 0.008), "**expresses affection**" (p = 0.014), "**social smile**" (p = 0.047), "**offers or shares something**" (p = 0.046), "**performs social productions**" (p = 0.005), and "**symbolic play**" (p = 0.005).

Additionally, although some skills did not reach statistical significance, an improvement was observed in the mean post-intervention score for some skills, as seen in Table 2. For example, "engaged play" showed an increase of Δ = +0.78 (p = 0.053), close to the significance threshold, and "responds to 'no'" showed Δ = +0.44 (p = 0.095). An improvement was also observed in "respects turns" (p = 0.053), pointing to a trend of evolution. These results suggest relevant clinical gains, even if not statistically confirmed, especially considering the sample size.

**Table 2:** Social skills with a mean increase or positive trend post-intervention, not statistically significant (p > 0.05)

**Skill**	**1. Before, N = 23**	**2. After, N = 23**	**p-value**	**Notes**
**Engaged play**			**0.053**	**Substantial increase**
Mean (SD)	1.22 (1.13)	2.00 (1.45)		
Median [IQI]	1.00 [0.00; 2.00]	2.00 [1.00; 3.00]		
**Responds to "no"**			**0.095**	**Mild improvement**
Mean (SD)	1.52 (1.16)	1.96 (1.07)		
Median [IQI]	2.00 [0.50; 2.00]	2.00 [2.00; 2.00]		
**Respects turns**			**0.095**	**Trend towards improvement**
Mean (SD)	0.26 (0.86)	1.00 (1.60)		
Median [IQI]	0.00 [0.00; 0.00]	0.00 [0.00; 2.00]		

**Caption**: SD – Standard Deviation; IQI – Amplitude Interquartile

On the other hand, "asks questions" (p = 0.371), "takes initiative to carry out activities" (p = 0.350), and "does not throw tantrums" (p = 0.638) did not show significant changes or a relevant average increase after the intervention, suggesting that such skills may require more intervention time or additional strategies for effective development, as seen in Table 3 below.

**Table 3:** Skills with no relevant average increase and no statistical significance (p > 0.05)

**Skill**	**1. Before, N = 23**	**2. After, N = 23**	**p-value**	**Notes**
**Starts activities**			**0.35**	**Slight increase**
Mean (SD)	2.13 (1.52)	2.43 (1.24)		
Median [IQI]	2.00 [0.50; 3.00]	2.00 [1.50; 3.50]		
**Does not throw tantrums**			**0.638**	**Subtle difference**
Mean (SD)	2.17 (1.53)	2.43 (1.38)		
Median [IQI]	2.00 [1.00; 4.00]	2.00 [2.00; 4.00]		
**Asks questions**			**0.371**	**Initial average = 0**
Mean (SD)	0.00 (0.00)	0.22 (0.74)		
Median [IQI]	0.00 [0.00; 0.00]	0.00 [0.00; 0.00]		

**Caption**: SD – Standard Deviation; IQI – Amplitude Interquartile

## DISCUSSION

The study findings indicate that intervention with the DHACA method promoted significant gains in the development of social skills in children with ASD. The comparative analysis of scores for behaviors and social communication skills, measured with ACOTEA-R before and after the intervention, showed significant changes in the socio-communicative repertoire of the participants.

The shared attention skill, frequently described as deficient in individuals with ASD, stands out with statistically significant improvement (p = 0.003). This skill represents an important milestone in communicative development, as it involves the ability to coordinate attention between the interlocutor and an object or event, allowing the attribution of intentionality to the other's action and enabling joint reference experiences, essential for the emergence of more complex communicative behaviors^(16)^.

Another relevant result refers to the increase in eye contact (p = 0.001), an important indicator of social skills and interactional engagement. Eye contact is directly related to attention regulation in in-person interactions, assisting in the coordination of shared attention and social reciprocity, aspects frequently compromised in the clinical profile of autism^(17)^.

The intervention also resulted in statistically significant advances in other skills, such as responding to name (p = 0.003), understanding and executing simple commands (p = 0.003), performing social productions (p = 0.005), imitation (p = 0.008), expressing affection (p = 0.014), social smile (p = 0.047), and respecting turns (p = 0.053). The improvement in these indicators reinforces the potential of the DHACA method in expanding social responsiveness and the emergence of pro-communicative behaviors, frequently impaired in children with ASD^(10,18)^.

Statistically significant advances were found in the ability to engage in symbolic play (p = 0.005), which teaches rules of coexistence, develops social skills such as sharing, taking turns, and cooperating, and fosters symbolic thinking. In this direction, the ability to offer or share something (p = 0.046) also had a significant result, relevant for engagement and the formation of future social bonds^(19,20)^.

Although some skills did not reach statistical significance, their average scores increased after the intervention, suggesting a trend of functional improvement. Among these, the following stand out: engaged play (Δ = +0.78; p = 0.053), responding to “no” (Δ = +0.44; p = 0.095), taking initiative in carrying out activities (Δ = +0.39; p = 0.350), asking questions (Δ = +0.26; p = 0.371), and not throwing tantrums (Δ = +0.13; p = 0.638). These findings, although not statistically significant, may represent relevant clinical gains, particularly in the context of early intervention, and reinforce the need for studies with a greater sample size and longitudinal follow-up, which are limitations of this study.

It is worth highlighting that the study has limitations that should be considered when interpreting the findings: the parents received regular guidance, but their adherence was not monitored, nor was its impact controlled, which may restrict the understanding of the observed gains. Also, it was not possible to control the use of the AAC system of the DHACA® method by partners at home. In addition, the study did not record the level of support of the spectrum foreseen by the DSM-5, which limited the possibility of analysis between different support level profiles. These aspects should be considered in future investigations, systematically monitoring relevant family and clinical variables.

The study results point to the need for longer-lasting interventions to help children improve the development of social skills. Future research should explore AAC intervention with the DHACA method and a larger sample of children with ASD.

## CONCLUSION

The findings suggest improvements in the development of social skills in children with ASD associated with intervention using the DHACA® method, indicating potential clinical benefits. These results offer preliminary evidence pointing to the value of the method as a therapeutic support aimed at expanding the social and communicative participation of children with ASD.
