# Supplementary material for: Clinical impact of the Development of Communication Skills in Autism (DHACA) on the development of social skills in children with autism
Source: Codas. 2026 Aug 3;38(5):e20250202. doi: 10.1590/2317-1782/e20250202en (PMC13445985; doi:10.1590/2317-1782/e20250202en)
Supplement: Translation

# Translation: Impacto clínico do método Desenvolvimento das Habilidades de Comunicação no Autismo (DHACA) no desenvolvimento de habilidades sociais em crianças com autismo

**DOI:** 10.1590/2317-1782/e20250202pt

**Authors:** Letícia Cristiny Arcanjo da Silva, Fádja Auxiliadora Alves e Silva, Rafaela Asfora Siqueira Campos Lima, Ana Cristina de Albuquerque Montenegro

**Affiliations:** 1 Departamento de Fonoaudiologia, Universidade Federal de Pernambuco – UFPE - Recife (PE), Brasil.; 2 Programa de Pós-Graduação em Saúde da Comunicação Humana, Departamento de Fonoaudiologia, Universidade Federal de Pernambuco –UFPE - Recife (PE), Brasil; 3 Departamento de Psicologia, Inclusão e Educação – DPSIE, Universidade Federal de Pernambuco – UFPE - Recife (PE), Brasil.

**Keywords:** Transtorno do Espectro do Autismo, Habilidades Sociais, Comunicação Aumentativa e Alternativa, Linguagem, Fonoaudiologia

## Abstract

**Objetivo:**

avaliar o impacto clínico do método DHACA® no desenvolvimento de habilidades sociais em crianças com TEA.

**Método:**

Estudo de intervenção, longitudinal e quantitativo, realizado com 23 crianças com TEA, entre 3 e 6 anos, não-verbais ou minimamente verbais. As sessões individuais de intervenção com o DHACA® ocorreram semanalmente durante 20 semanas. A avaliação das habilidades sociais foi realizada antes e após a intervenção, por meio do protocolo ACOTEA-R. Os dados foram analisados estatisticamente com o teste de Wilcoxon.

**Resultados:**

Houve melhora significativa (p < 0,05) em dez das 16 habilidades avaliadas, incluindo “responde pelo nome”, “contato visual”, “atenção compartilhada” e “brincar simbólico”. Outras habilidades, como “responde ao ‘não’” e “respeita turnos”, apresentaram tendência de melhora clínica, ainda que sem significância estatística. Apenas três habilidades não demonstraram evolução relevante.

**Conclusão:**

Os achados indicam melhorias nas habilidades sociais de crianças com TEA associadas ao uso do método DHACA®, contribuindo para maior autonomia e participação social. Os resultados reforçam seu potencial clínico como recurso terapêutico em contextos educacionais e de saúde.

## INTRODUÇÃO

O Transtorno do Espectro do Autismo (TEA) é um distúrbio do neurodesenvolvimento caracterizado por déficits persistentes na comunicação, dificuldades na interação social e padrões de comportamento restritos e repetitivos^(1)^. Essas características podem variar em gravidade e funcionalidade, manifestando-se desde os primeiros anos de vida^(2)^. Dados recentes do Center for Disease Control and Prevention (CDC) indicam que o TEA afeta aproximadamente uma em cada 36 crianças de 8 anos em 11 estados norte-americanos^(3)^, evidenciando um aumento significativo em relação aos levantamentos anteriores^(4)^. Essa crescente prevalência reforça a necessidade de intervenções eficazes que visem minimizar as barreiras comunicacionais e promover a inclusão social de indivíduos com TEA.

As dificuldades no funcionamento social são uma das principais características definidoras do autismo^(5)^. A percepção social, um dos componentes fundamentais das habilidades sociais, refere-se à capacidade de interpretar e atribuir significado a estímulos sociais, como expressões faciais, tom de voz e comportamentos. Indivíduos com TEA apresentam déficits na percepção social, o que compromete o desenvolvimento de habilidades essenciais para a interação social. Além disso, há prejuízos nas habilidades cognitivas e na reciprocidade social, impactando significativamente a qualidade de vida desses indivíduos^(6)^.

Del Prette e Del Prette^(7)^ definem habilidades sociais como um conjunto de comportamentos aprendidos que possibilitam ao indivíduo interagir de forma eficaz em contextos sociais, promovendo aceitação e inclusão. Estudos indicam que algumas das principais habilidades sociais incluem sorrir e manter contato visual, iniciar e manter interações, compartilhar atenção, inferir interesses e adotar a perspectiva dos outros^(5)^. A promoção dessas habilidades pode ser facilitada por meio de abordagens inovadoras, como a Comunicação Aumentativa e Alternativa (CAA), que visa ampliar as possibilidades comunicativas de indivíduos com dificuldades expressivas^(8)^.

A CAA tem sido amplamente estudada como um recurso eficaz para promover a comunicação e a inclusão social de indivíduos com TEA^(9)^. Segundo a American Speech-Language-Hearing Association (ASHA), a CAA compreende técnicas, recursos e estratégias que facilitam a comunicação de pessoas com Necessidades Complexas de Comunicação (NCC)^(2)^. Entre os benefícios descritos na literatura, destacam-se a melhoria na expressão facial e corporal, o desenvolvimento da linguagem oral, o aprimoramento da linguagem não verbal e a ampliação da participação social^(9)^. Esses fatores contribuem para a redução da frustração, minimização de comportamentos inadequados e fortalecimento das interações interpessoais^(9)^.

O método Desenvolvimento das Habilidades de Comunicação no Autismo (DHACA®) é uma abordagem inovadora dentro da CAA, baseada na Teoria Sociopragmática ou Teoria de Aquisição da Linguagem baseada no uso, que enfatiza o papel da interação social no desenvolvimento da comunicação. Esse método utiliza como recurso um livro de comunicação com pictogramas que representam o vocabulário essencial e acessório, organizados em categorias lexicais, para favorecer a aquisição e o uso funcional da linguagem. O DHACA® é composto por cinco habilidades que estimulam o desenvolvimento da comunicação funcional por meio de estratégias como o uso da modelagem e dicas físicas, visuais e verbais. Além disso, sua abordagem fundamenta-se no interesse individual da criança, tornando a aprendizagem mais significativa e motivadora. A implementação do DHACA® tem demonstrado potencial para ampliar as habilidades comunicativas e sociais de crianças com TEA, proporcionando maior autonomia, participação ativa na interação social e qualidade de vida^(10)^.

Dado o aumento da prevalência do TEA e a necessidade de estratégias eficazes para o desenvolvimento de habilidades sociais, torna-se essencial investigar o impacto do método DHACA® na promoção dessas competências. A escassez de estudos que avaliem a relação entre a aplicação do DHACA® e o aprimoramento das habilidades sociais em crianças com TEA reforça a necessidade de pesquisas que analisem seus benefícios. Assim, este estudo tem por objetivo avaliar os efeitos do método DHACA no desenvolvimento de habilidades sociais em crianças com TEA.

## MÉTODO

Este estudo de natureza longitudinal e abordagem quantitativa, do tipo intervenção, foi realizado na Clínica-Escola de Fonoaudiologia Professor Fábio Lessa, vinculada ao Departamento de Fonoaudiologia da Universidade Federal de Pernambuco (UFPE). O projeto foi aprovado pelo Comitê de Ética em Pesquisa com Seres Humanos sob protocolos n.2.106.800 e n. 6.617.543, respeitando os preceitos éticos estabelecidos na Resolução nº 466/2012 do Conselho Nacional de Saúde.

Participaram da pesquisa 23 crianças com diagnóstico clínico de Transtorno do Espectro do Autismo (TEA), participantes do projeto de extensão e pesquisa Autismo Comunica nos anos de 2022 e 2023, sendo quatro do sexo feminino e 19 do sexo masculino. Dentre essas, oito não-falantes e 15 minimamente falantes, encaminhadas pelo Sistema Único de Saúde (SUS) à Clínica-Escola. Os critérios de inclusão foram: idade entre três e seis anos, com diagnóstico de TEA segundo os critérios estabelecidos pela Associação Americana de Psiquiatria no DSM-5 (2015), que não estivessem em intervenção fonoaudiológica externa concomitante. Foram excluídas crianças com perfil comunicativo predominantemente falante, bem como aquelas com comorbidades associadas ao TEA, como síndromes genéticas, malformações, alterações neurológicas e deficiências físicas, intelectuais, auditivas ou visuais.

Os responsáveis legais das crianças foram convidados a participar e, após o esclarecimento sobre os objetivos e procedimentos do estudo, assinaram o Termo de Consentimento Livre e Esclarecido (TCLE). Posteriormente, foi realizada uma entrevista de anamnese clínica e aplicação de um questionário sociodemográfico, a fim de caracterizar os participantes e registrar o histórico clínico e familiar. Quanto ao perfil parental de escolaridade, 20 responsáveis tinham o Ensino Médio e três tinham o Ensino Fundamental.

As crianças foram avaliadas antes e após a intervenção fonoaudiológica por meio do protocolo ACOTEA-R (Avaliação da Comunicação de Crianças com Autismo)^(11)^, utilizando atividades lúdicas e interativas em ambiente terapêutico. O ACOTEA-R permite a observação de 36 habilidades sociais e comunicativas, distribuídas entre as seguintes seções: Comunicação Expressiva, Comunicação Receptiva e Comportamento Social. Dentre as habilidades do instrumento, 16 foram selecionadas para análise, privilegiando itens cuja natureza é predominantemente social. Não foram incluídas habilidades relacionadas principalmente à comunicação, às questões comportamentais, por não constituírem indicadores diretos de sociabilidade. A decisão metodológica baseou-se em modelos desenvolvimentistas que descrevem uma hierarquia de competências sociais iniciais, como atenção compartilhada, imitação, brincar engajado, consideradas pré-requisitos para a emergência de repertórios sociais mais complexos^(11-13)^.

Assim sendo, as habilidades escolhidas foram: oferece ou compartilha algo (P11), realiza produções sociais (P14), faz perguntas (P15), respeita turnos (P19), não realiza birras (P20), sorriso social (P21), responde pelo nome (P22), responde ao “não” (P24), compreende e executa comandos simples (P25), imitação (P26), atenção compartilhada (P27), expressa afeto (P28), contato visual (P29), tem iniciativa de realizar atividades (P32), brincar simbólico (P35) e brincar engajado (P36). A avaliação com o ACOTEA-R foi registrada por meio de uma gravação previamente autorizada pelos responsáveis por meio de Termos de Autorização e Uso de Imagem. O preenchimento de respostas do instrumento tanto na fase pré quanto na pós intervenção, foi realizado por um avaliador cego à condição.

A intervenção com o método DHACA® foi conduzida em 20 sessões individuais, semanais, com duração de 45 minutos cada. As atividades foram planejadas minuciosamente de forma lúdica, a partir de brincadeiras estruturadas e semiestruturadas, respeitando as preferências individuais de cada criança, previamente identificadas por meio da Avaliação Indireta de Preferência^(10)^ (AIP). O instrumento, respondido pelo responsável, tem como objetivo identificar as preferências da criança em diferentes domínios, incluindo escolhas alimentares, interações sociais, estímulos sensoriais, além de brinquedos, atividades e objetos de interesse. As estratégias terapêuticas envolveram o uso de dicas, sejam elas de natureza física (dica física parcial ou total), visual (gestos direcionadores e recursos visuais) ou verbal. Esses elementos foram incorporados com a finalidade de tornar as propostas mais motivadoras, facilitar a assimilação das tarefas e incentivar respostas mais acertadas por parte das crianças^(14,15)^. A redução gradativa das dicas foi aplicada de forma individualizada, conforme o desempenho de cada participante, respeitando seu ritmo de aprendizagem.

As intervenções foram conduzidas por estudantes de graduação e pós-graduação em Fonoaudiologia, previamente capacitados no início do ano letivo e continuamente supervisionados ao longo do processo por docentes do grupo de pesquisa e extensão “Autismo Comunica”. A supervisão ocorreu em tempo real por meio de espelho unidirecional, além de encontros regulares de discussão de casos e revisão de estratégias terapêuticas.

Os responsáveis pelas crianças participaram de orientações individuais semanais durante ou após a sessão de intervenção e a cada dois meses, participavam de reuniões de orientação parental com duração estimada de três horas, nas quais eram discutidas as bases teóricas das habilidades sociais e comunicativas trabalhadas, estratégias práticas para uso do livro DHACA® no cotidiano e momentos de escuta, partilha e esclarecimento de dúvidas. Essa participação visou ampliar o envolvimento da família no processo terapêutico, possibilitando continuidade e reforço das intervenções no ambiente familiar.

Os dados coletados foram organizados em planilhas digitais no Microsoft Excel e, posteriormente, submetidos à análise estatística. As habilidades sociais e comunicativas foram analisadas por meio de medidas descritivas (média, desvio padrão, mediana e amplitude interquartil). Como os dados não apresentaram distribuição normal, verificada pelo teste de Shapiro-Wilk, foi aplicado o teste de Wilcoxon com correção de continuidade para comparação dos escores entre os momentos pré e pós-intervenção. Esse teste é indicado para amostras emparelhadas com distribuição assimétrica, como no caso do presente estudo.

## RESULTADOS

Os resultados dessa pesquisa apontam que a intervenção com a Comunicação Aumentativa e Alternativa (CAA) por meio do método DHACA favoreceu o desenvolvimento de habilidades sociais dos participantes. Houve melhora estatisticamente significativa evidenciada pelos valores de *p* abaixo de 0,05, em diversos itens relacionados a habilidades sociais e comunicativas, conforme visto na tabela 1, a seguir:

**Tabela 1:** Escore das habilidades sociais do protocolo ACOTEA pré e pós intervenção em crianças com autismo com melhora estatisticamente significativa (p < 0,05)

**Habilidade**	**1.Antes, N=23**	**2.Depois, N=23**	**Valor p1**
**Responde pelo nome**			**0,003**
Média (DP)	1,52 (0,95)	2,52 (0,95)	
Mediana [AIQ]	2,00 [1,00, 2,00]	2,00 [2,00, 3,00]	
**Contato Visual**			**0,001**
Média (DP)	1,91 (0,95)	2,74 (0,96)	
Mediana [AIQ]	2,00 [1,50, 2,00]	3,00 [2,00, 3,00]	
**Atenção compartilhada**			**0,003**
Média (DP)	1,52 (0,79)	2,43 (1,20)	
Mediana [AIQ]	2,00 [1,00, 2,00]	3,00 [1,50, 3,00]	
**Compreende e executa comandos simples**			**0,003**
Média (DP)	1,74 (1,18)	2,78 (1,09)	
Mediana [AIQ]	2,00 [1,00, 3,00]	3,00 [2,00, 4,00]	
**Imitação**			**0,008**
Média (DP)	1,39 (1,03)	2,13 (1,22)	
Mediana [AIQ]	1,00 [1,00, 2,00]	2,00 [1,00, 3,00]	
**Expressa afeto**			**0,014**
Média (DP)	0,87 (1,06)	1,48 (1,38)	
Mediana [AIQ]	1,00 [0,00, 1,00]	1,00 [0,00, 3,00]	
**Brincar simbólico**			**0,005**
Média (DP)	1,35 (1,23)	2,26 (1,48)	
Mediana [AIQ]	1,00 [0,00, 2,00]	3,00 [1,00, 3,00]	
**Oferece ou compartilha algo**			**0,046**
Média (DP)	0,57 (0,79)	1,22 (1,41)	
Mediana [AIQ]	0,00 [0,00, 1,00]	0,00 [0,00, 2,50]	
**Sorriso social**			**0,047**
Média (DP)	2,00 (1,24)	2,48 (1,20)	
Mediana [AIQ]	2,00 [1,00, 2,50]	2,00 [2,00, 3,50]	
**Realiza produções sociais**			**0,005**
Média (DP)	0,48 (0,99)	1,52 (1,41)	
Mediana [AIQ]	0,00 [0,00, 0,00]	2,00 [0,00, 2,00]	

**Legenda:** DP = Desvio Padrão; AIQ = Amplitude Interquartil

Entre as habilidades com diferenças significativas, destacam-se: “**responde pelo nome**” (*p* = 0,003), “**contato visual**” (*p* = 0,001), “**atenção compartilhada**” (*p* = 0,003), “**compreende e executa comando simples**” (*p* = 0,003), “**imitação**” (*p* = 0,008), “**expressa afeto**” (*p* = 0,014), “**sorriso social**” (*p* = 0,047), “**oferece ou compartilha algo**” (*p* = 0,046), “r**ealiza produções sociais**” (*p* = 0,005) e “**brincar simbólico**” (*p* = 0,005).

Adicionalmente, embora algumas habilidades não tenham atingido significância estatística, observou-se melhora no escore médio pós-intervenção de algumas habilidades, conforme visto na tabela 2. Por exemplo, “brincar engajado” apresentou um aumento de Δ = +0,78 (*p* = 0,053), próximo do limiar de significância, e “responde ao ‘não’” apresentou Δ = +0,44 (*p* = 0,095). Também se observou melhora em “respeita turnos” (*p* = 0,053), apontando para uma tendência de evolução. Tais resultados sugerem ganhos clínicos relevantes, mesmo que não estatisticamente confirmados, especialmente considerando o tamanho da amostra.

**Tabela 2:** Habilidades sociais com aumento médio ou tendência positiva pós-intervenção, não estatisticamente significativas (p > 0,05)

**Habilidade**	**1. Antes, N=23**	**2. Depois, N=23**	**p-valor**	**Observações**
**Brincar engajado**			**0,053**	**Aumento expressivo**
Média (DP)	1,22 (1,13)	2,00 (1,45)		
Mediana [AIQ]	1,00 [0,00; 2,00]	2,00 [1,00; 3,00]		
**Responde ao "não"**			**0,095**	**Melhora leve**
Média (DP)	1,52 (1,16)	1,96 (1,07)		
Mediana [AIQ]	2,00 [0,50; 2,00]	2,00 [2,00; 2,00]		
**Respeita turnos**			**0,095**	**Tendência de melhora**
Média (DP)	0,26 (0,86)	1,00 (1,60)		
Mediana [AIQ]	0,00 [0,00; 0,00]	0,00 [0,00; 2,00]		

**Legenda:** DP = Desvio Padrão; AIQ = Amplitude Interquartil

Por outro lado, foi observado que as habilidades “faz perguntas” (*p* = 0,371), “tem iniciativa de realizar atividades” (*p* = 0,350) e “não realiza birras” (*p* = 0,638) não apresentaram mudanças significativas após a intervenção ou aumento médio relevante, sugerindo que tais competências podem demandar mais tempo de intervenção ou estratégias adicionais para desenvolvimento efetivo, conforme visto na tabela 3, a seguir:

**Tabela 3:** Habilidades sem aumento médio relevante e sem significância estatística (p > 0,05)

**Habilidade**	**1. Antes, N=23**	**2. Depois, N=23**	**p-valor**	**Observações**
**Inicia atividades**			**0,35**	**Aumento pequeno**
Média (DP)	2,13 (1,52)	2,43 (1,24)		
Mediana [AIQ]	2,00 [0,50; 3,00]	2,00 [1,50; 3,50]		
**Não realiza birras**			**0,638**	**Diferença sutil**
Média (DP)	2,17 (1,53)	2,43 (1,38)		
Mediana [AIQ]	2,00 [1,00; 4,00]	2,00 [2,00; 4,00]		
**Faz perguntas**			**0,371**	**Média inicial=0**
Média (DP)	0,00 (0,00)	0,22 (0,74)		
Mediana [AIQ]	0,00 [0,00; 0,00]	0,00 [0,00; 0,00]		

**Legenda:** DP = Desvio Padrão; AIQ = Amplitude Interquartil

## DISCUSSÃO

Os achados deste estudo indicam que a intervenção com o método DHACA promoveu ganhos significativos no desenvolvimento de habilidades sociais em crianças com Transtorno do Espectro Autista (TEA). A análise comparativa dos escores de comportamentos e habilidades de comunicação social, mensurados por meio do instrumento ACOTEA-R, antes e após a intervenção, evidenciou modificações expressivas no repertório sociocomunicativo dos participantes.

Destaca-se a habilidade de atenção compartilhada, frequentemente descrita como deficitária em indivíduos com TEA, que apresentou evolução estatisticamente significativa (p = 0,003). Essa habilidade representa um marco importante do desenvolvimento comunicativo, pois envolve a capacidade de coordenar a atenção entre o interlocutor e um objeto ou evento, permitindo a atribuição de intencionalidade à ação do outro e viabilizando experiências de referência conjunta, essenciais para o surgimento de comportamentos comunicativos mais complexos^(16)^.

Outro resultado relevante refere-se ao aumento no contato visual (p = 0,001), indicador importante de habilidade social e engajamento interacional. O contato visual está diretamente relacionado à regulação da atenção no contexto de interações face a face, auxiliando a coordenação da atenção compartilhada, e assim, a reciprocidade social, aspectos frequentemente comprometidos no perfil clínico do autismo^(17)^.

A intervenção também resultou em avanços estatisticamente significativos em outras habilidades, tais como: responder pelo nome (p = 0,003), compreender e executar comandos simples (p = 0,003), realizar produções sociais (p = 0,005), imitação (p = 0,008), expressar afeto (p = 0,014), sorriso social (p = 0,047) e respeitar turnos (p = 0,053). A melhora nesses indicadores reforça o potencial do método DHACA na ampliação da responsividade social e na emergência de comportamentos pró-comunicativos, frequentemente prejudicados em crianças com TEA^(10,18)^.

Constatou-se avanços estatisticamente significativos na habilidade de brincar simbólico (p = 0,005), o que proporciona a aprendizagem de regras de convivência e o desenvolvimento de habilidades sociais como compartilhar, revezar, cooperar, fomentando também o pensamento simbólico. Nessa direção, a habilidade de oferecer ou compartilhar algo (p = 0,046) também apresentou resultado significativo, relevante para o engajamento e formação de vínculos sociais futuros^(19,20)^.

Ainda que algumas habilidades não tenham atingido significância estatística, observou-se incremento nos escores médios no pós-teste, sugerindo uma tendência de melhora funcional. Entre elas, destacam-se: brincar engajado (Δ = +0,78; p = 0,053), responder ao “não” (Δ = +0,44; p = 0,095), ter iniciativa de realizar atividades (Δ = +0,39; p = 0,350), fazer perguntas (Δ = +0,26; p = 0,371) e não realizar birras (Δ = +0,13; p = 0,638). Esses achados, embora não estatisticamente significativos, podem representar ganhos clínicos relevantes, particularmente no contexto da intervenção precoce, e reforçam a necessidade de estudos com maior poder amostral e acompanhamento longitudinal, sendo estas limitações deste estudo.

Vale destacar que o estudo apresenta limitações que devem ser consideradas na interpretação dos achados: a orientação parental, embora realizada regularmente, não condicionou a um monitoramento de adesão parental, nem seu impacto controlado, o que pode restringir a compreensão dos ganhos observados; não foi possível controlar o uso do sistema de CAA do método DHACA® pelos parceiros em domicílio. Além disso, o nível de suporte do espectro previsto pelo DSM-5 não foi registrado, o que limitou a possibilidade de análise entre diferentes perfis de nível de suporte. Esses aspectos devem ser contemplados em futuras investigações, com monitoramento sistemático das variáveis familiares e clínicas relevantes.

Os resultados deste estudo apontam para a necessidade de intervenções mais duradouras para auxiliar as crianças a aprimorar o desenvolvimento de habilidades sociais. Pesquisas futuras devem explorar uma intervenção em Comunicação Aumentativa e Alternativa, com o método DHACA, com uma amostra maior de crianças com TEA.

## CONCLUSÃO

Os achados sugerem que houve melhorias no desenvolvimento de habilidades sociais em crianças autistas associadas à intervenção com o método DHACA®, indicando potenciais benefícios clínicos. Tais resultados oferecem evidências preliminares que apontam para o valor do método como suporte terapêutico voltado à ampliação da participação social e comunicativa de crianças com TEA.
